# Development and internal validation of a nomogram for predicting referral or hospitalization risk in children with community-acquired influenza

**DOI:** 10.3389/fped.2026.1779925

**Published:** 2026-04-10

**Authors:** Xiaoman Cai, Jiabin Yang, Shiyou Luo, Mufan Chen, Yaoting Su, Ailing Zhang, Bin Wang, Xueying Meng

**Affiliations:** Longdong Community Health Service Center, Longgang Central Hospital, Shenzhen, China

**Keywords:** children, hospitalization, influenza, nomogram, predictive model, referral

## Abstract

**Background:**

To develop and internally validate a nomogram for predicting referral or hospitalization risk in children with community-acquired influenza based on clinical indicators, thereby providing primary healthcare institutions with evidence-based decision-making tools.

**Methods:**

Clinical data were prospectively collected from children aged 6 months to 6 years diagnosed with influenza at Longdong Community Health Service Center, Longgang Central Hospital, Shenzhen, between May 2024 and October 2025. Independent risk factors were identified through univariate and multivariable logistic regression analyses to construct nomograms. Model performance was evaluated using receiver operating characteristic (ROC) curves, calibration plots, decision curve analysis (DCA), and Bootstrap internal validation. To address potential bias from clinical decision-related variables, a sensitivity analysis was further performed to verify the model's robustness.

**Results:**

A total of 388 children were finally enrolled in this study, among whom 49 (12.6%) required referral or hospitalization. Multivariable analysis revealed that lack of influenza vaccination, antibiotic usage, sore throat, myalgia, gastrointestinal symptoms, elevated C-reactive protein levels, and increased frequency of medical visits were independent risk factors (*P* < 0.05). The nomogram constructed based on these seven factors demonstrated an area under the curve (AUC) of 0.95 (95% CI: 0.92–0.98), with accuracy of 0.88, sensitivity of 0.92, and specificity of 0.88. Calibration curves indicated excellent model fit (Hosmer-Lemeshow test *P* = 0.862), while DCA demonstrated significant clinical net benefit. Bootstrap validation confirmed robust model stability, and sensitivity analysis excluding bias-prone variables further validated the reliability of the model's core conclusions.

**Conclusion:**

This nomogram, utilizing readily accessible clinical parameters, exhibited superior predictive performance for referral or hospitalization risk assessment in children with community-acquired influenza. It provided an intuitive and practical tool for precise patient triage in primary care settings, potentially reducing healthcare resource wastage.

## 1 Introduction

Community-acquired influenza is a prevalent acute respiratory infectious disease among children, exhibiting distinctive seasonal epidemic characteristics and extensive transmission patterns (1). Globally, influenza causes substantial pediatric outpatient visits and hospitalizations annually, with healthcare resources frequently strained during peak influenza seasons. The rapid mutation and transmission of influenza viruses present ongoing public health challenges, while children, as a vulnerable population, exhibit significantly higher infection rates and complication risks compared to adults. Therefore, early intervention and risk stratification management for pediatric influenza are of paramount importance (2).

The health hazards of influenza in children are manifested not only in acute-phase symptoms such as high fever, respiratory symptoms, and systemic discomfort, but may also lead to serious complications including pneumonia, myocarditis, and encephalitis, potentially resulting in death (3). Infants and young children, as well as children with underlying diseases or immunocompromised conditions, are at particularly high risk (4). Current treatment for influenza primarily consists of symptomatic supportive care and antiviral medications, but the efficacy is closely related to timing. Early identification of high-risk children and timely referral or hospitalization intervention are key to improving prognosis, reducing severe illness rates, and decreasing healthcare burden (5). Therefore, establishing scientific and rapid risk assessment tools to help community and primary healthcare institutions properly triage children has important clinical necessity.

Currently, several studies have explored risk factors for severe illness or ICU referral in children with influenza, such as young age, comorbid underlying diseases, and severity of symptoms. Based on these findings, several clinical scoring systems have been developed (6–8). For example, Sun et al. (9) constructed a nomogram model based on six independent risk factors (elevated peripheral white blood cells, increased large platelet ratio, decreased mean platelet volume, reduced complement C3, elevated globulin, and decreased total IgM), which demonstrated extremely high accuracy in predicting ICU referral risk for hospitalized children with influenza (C-index 0.970, AUC 0.966 in training set, 0.919 in validation set). Additionally, Cheong et al. (10)'s study found that the PRISM III scoring system also demonstrated high sensitivity and specificity in predicting mortality risk in children with influenza. Existing risk prediction models for children with influenza mainly focus on ICU referral or mortality risk among hospitalized children, and most of the included indicators are complex laboratory tests that are difficult to implement in primary healthcare settings. There is still a gap in risk stratification tools for referral or hospitalization of community-based children with influenza aged 6 months to 6 years, leaving primary care physicians without scientific decision-making evidence. This leads to delayed referrals for some high-risk patients or overtreatment of low-risk patients.

This study aimed to construct and internally validate a risk prediction model for referral or hospitalization of community-based children with influenza, based on readily available clinical indicators and utilizing the SHAP interpretation framework and nomogram visualization tools. By identifying key risk factors and achieving individualized visual risk assessment, this study expected to provide primary healthcare professionals with an intuitive, reliable, and easy-to-operate decision support tool. This would promote hierarchical management of children and rational allocation of medical resources, ultimately achieving the goals of improving diagnostic efficiency and patient outcomes.

## 2 Materials and methods

### 2.1 Study design

**Figure 1:**
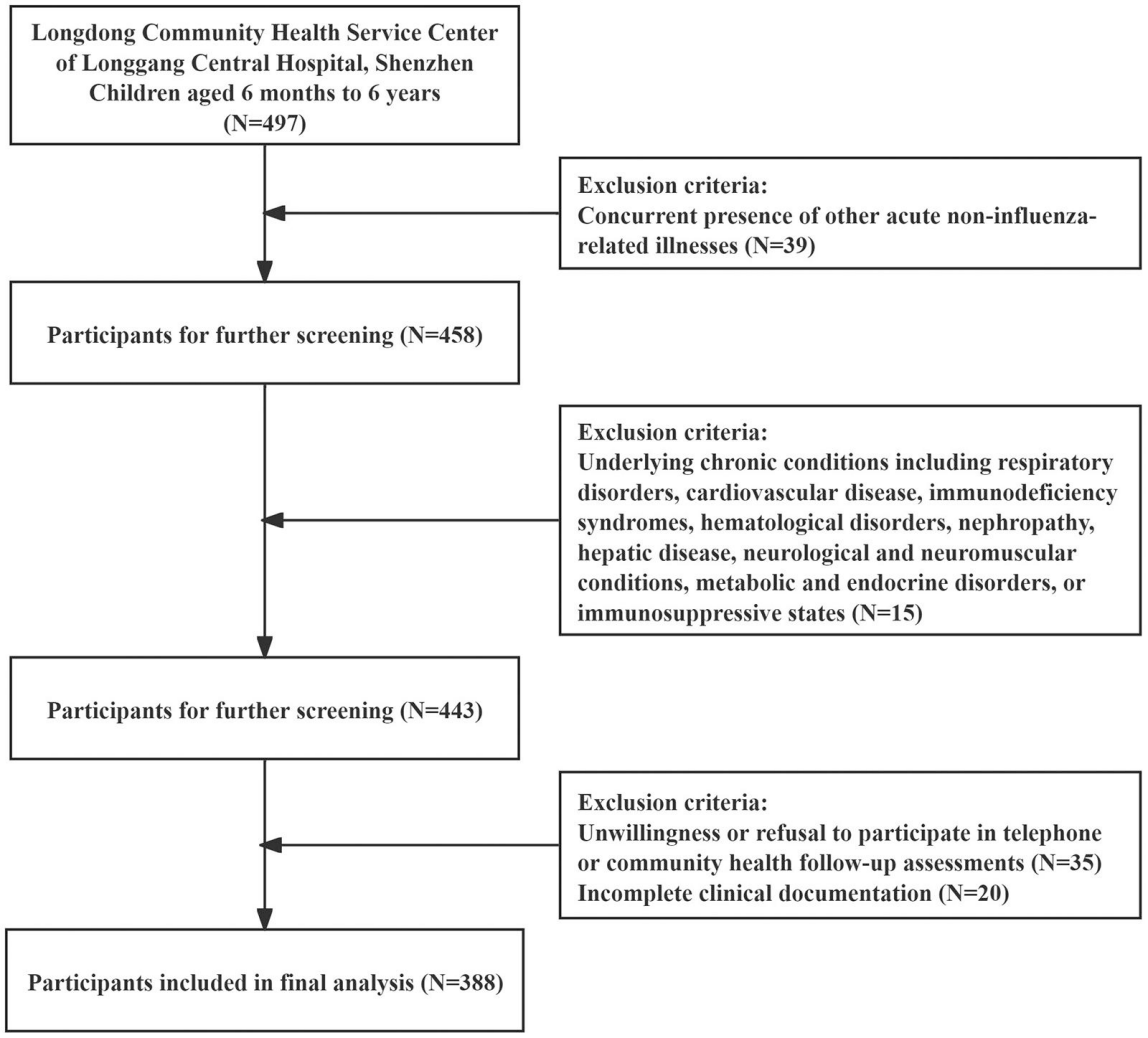
Flowchart of participant enrollment and study procedure. This flowchart details the screening, inclusion and exclusion process of children with community-acquired influenza enrolled in this prospective cohort study, as well as the follow-up and primary endpoint assessment workflow.

This was a single-center prospective cohort study. Clinical data of children aged 6 months to 6 years who visited Longdong Community Health Service Center of Longgang Central Hospital, Shenzhen, for influenza-like illness were prospectively collected from May 15, 2024, to October 16, 2025. Nasopharyngeal swab specimens were collected, and children confirmed with influenza through influenza A or B virus antigen testing were included in the study (Figure 1).

Inclusion criteria: (1) Meeting the diagnostic criteria for influenza in the 2020 Expert Consensus on Diagnosis and Treatment of Influenza in Children; (2) Age 6 months to 6 years; (3) Guardians voluntarily signed written informed consent.

Exclusion criteria: (1) Concurrent presence of other acute non-influenza-related illnesses; (2) Underlying chronic conditions including respiratory disorders, cardiovascular disease, immunodeficiency syndromes, hematological disorders, nephropathy, hepatic disease, neurological and neuromuscular conditions, metabolic and endocrine disorders, or immunosuppressive states; (3) Unwillingness or refusal to participate in telephone or community health follow-up assessments; (4) Incomplete clinical documentation.

This study was approved by the Ethics Committee of Shenzhen Longgang Central Hospital (Ethics approval number: 2024ECPJ026), with informed consent obtained from all guardians of pediatric participants.

### 2.2 Influenza viral antigen detection

Detection was performed using the influenza A or B virus antigen detection kit (colloidal gold method, Hangzhou Biotest Biotech Co., Ltd., Hangzhou, Zhejiang, China). A standardized nasopharyngeal swab collection method was employed: the cotton swab was completely inserted into the nasal cavity, rubbed against the nasal turbinates several times to collect mucosal epithelium. The cotton swab with the collected sample was then immersed in the sample extraction buffer in the collection tube and stirred, squeezed several times, then removed. The extracted liquid served as the test sample. 100 μL of the sample was dropped onto the sample well of the test plate, and results were observed after 15 min.

### 2.3 Data collection

General information of children included age (in months), gender (male/female), and preschool education history (yes/no); Clinical manifestations included body temperature (℃), duration of fever resolution (days), cough (yes/no), nasal congestion (yes/no), rhinorrhea (yes/no), sore throat (yes/no), muscle aches (yes/no), and gastrointestinal symptoms (yes/no, defined as the presence of at least one of vomiting, diarrhea, or abdominal pain); Laboratory tests (peripheral blood collected at the time of visit) included white blood cell count, neutrophil percentage and absolute value, lymphocyte percentage and absolute value, hemoglobin, platelet count, and C-reactive protein (CRP, with CRP > 10 mg/L defined as abnormal); Treatment details included influenza vaccination history (defined as vaccinated if immunized within 1 year prior to the visit, and unvaccinated if immunized ≥1 year ago or never immunized), use of antiviral medications (yes/no), and antibiotic use (yes/no); Healthcare utilization included number of medical visits (times) and outpatient costs (yuan). The timing of measurement for all variables was clearly specified: ① Baseline variables (obtained at the initial visit): history of influenza vaccination, sore throat, myalgia, gastrointestinal symptoms, C-reactive protein level, etc.; ② Post-baseline variables (generated during subsequent clinical care): antibiotic use, number of hospital/outpatient visits, outpatient expenses, etc.

### 2.4 Follow-up

Follow-up was conducted 7–10 days later through telephone calls or community health center visits to collect information on time to fever resolution, time to cough resolution, influenza-related hospitalizations, use of antiviral medications, complications, number of medical visits, and direct medical costs.

### 2.5 Outcome definition

The primary outcome was the need for referral or hospitalization, defined as: (1) referral to a higher-level hospital within 48 h after community clinic visit; (2) physician assessment recommending hospitalization with actual hospitalization of the child (excluding cases where guardians refused hospitalization).

### 2.6 Statistical analysis

Statistical analysis was performed using SPSS 30.0 (IBM Corp., Armonk, NY, USA) and R software version 4.4.3 (R Foundation for Statistical Computing, Vienna, Austria). All covariates had missing data proportions less than 20%, which were handled using five repeated multiple imputations, with the Multiple Imputation by Chained Equations (MICE) method employed to address missing data across repeated datasets. We provide dedicated tables in the Supplementary Materials, including: (Supplementary Table S1) the missing rate (percentage) of each variable in the model; (Supplementary Table S2) the differential analysis of the original dataset and the imputed dataset. All continuous variables were subjected to the Shapiro–Wilk normality test, which revealed non-normal distributions. Consequently, data were presented as median with interquartile range [M (Q_1_, Q_3_)], and inter-group comparisons were conducted using the Wilcoxon rank-sum test. Categorical variables were presented as frequencies and percentages, with comparisons performed using chi-square test or Fisher's exact test as appropriate. Two-sided *P* values < 0.05 were considered statistically significant.

Statistically significant predictive factors were identified through multivariable logistic regression to construct a nomogram prediction model. We adopted the following variable selection strategy: (1) First, we performed univariate logistic regression analysis for all collected variables, and incorporated variables with *P* < 0.05 into the candidate predictors; (2) Combined with clinical relevance (e.g., excluding variables with no clear association with the outcome), we conducted multivariate logistic regression analysis for the candidate variables, and included variables with *P* < 0.05 in the final nomogram model; (3) We assessed multicollinearity via the Variance Inflation Factor (VIF) test, where a VIF < 2 indicated no severe multicollinearity. Construction of Nomogram Based on Regression Coefficients from the Final Multivariable Logistic Regression Model: (1) We employed the lrm function from the R language rms package to fit the multivariable logistic regression model and obtained regression coefficients for each predictor variable. (2) We standardized all coefficients into scores within a 0–100 point range [Score = (Variable Coefficient/Maximum Coefficient) × 100]. (3) We calculated the total score as the sum of all predictor scores. (4) We converted the total score into predicted probability of referral/hospitalization through the logistic regression equation, and ultimately generated a visualization nomogram using the nomogram function from the rms package. The discriminative ability of the model was quantified by the area under the receiver operating characteristic (ROC) curve (AUC). The cutoff value was selected based on Youden index maximization (Youden index = sensitivity + specificity − 1), with the optimal cutoff value calculated via the pROC package in R. Calibration performance was validated through calibration curves combined with the Hosmer-Lemeshow (HL) test to assess model goodness-of-fit, and decision curve analysis (DCA) was used to evaluate the clinical utility of the prediction model. Internal validation of the nomogram model was performed using Bootstrap resampling with 1,000 iterations to enhance model stability.

### 2.7 Sensitivity analysis

To address the potential bias of variables affected by clinical decision-making or subsequent events, a sensitivity analysis was performed by constructing a novel baseline-only nomogram model that excluded antibiotic use and frequency of medical visits (variables with potential reverse causality or indication confounding). The novel model was developed using the same multivariable logistic regression method as the primary model, with nomogram visualization and performance evaluation consistent with the primary model: (1) Discriminative ability was assessed by AUC of ROC curve; (2) Calibration was verified by calibration curves and HL test; (3) Clinical utility was evaluated by DCA; (4) Internal validation was conducted via 1,000 iterations of Bootstrap resampling to confirm model stability.

## 3 Results

### 3.1 Comparison of clinical characteristics

A total of 388 children aged 6 months to 6 years with community-acquired influenza were prospectively enrolled in this study. After follow-up, 49 children (12.6%) met the primary endpoint and were assigned to the referral or hospitalization group, while the remaining 339 children (87.4%) were assigned to the non-referral or hospitalization group. The referral or hospitalization group showed significantly higher body temperature, fever duration, number of clinic visits, and outpatient costs compared to the non-referral or hospitalization group (*P* < 0.001). The prevalence of cough, runny nose, sore throat, muscle aches, gastrointestinal symptoms, and elevated C-reactive protein were all significantly higher in the referral or hospitalization group than in the non-referral or hospitalization group (*P* < 0.05). Meanwhile, the proportions of influenza vaccination and antiviral medication use were significantly lower in the referral or hospitalization group compared to the non-referral or hospitalization group, while the proportion of antibiotic use was significantly higher in the referral or hospitalization group (*P* < 0.001).

There were no statistically significant differences between the two groups in age distribution, routine blood parameters (white blood cell count, neutrophil percentage, lymphocyte percentage, absolute neutrophil count, absolute lymphocyte count, hemoglobin, platelet count), gender composition, education level, diagnosis type, and proportion of nasal congestion symptoms (*P* > 0.05) (Table 1).

**Table 1:** Comparison of baseline demographic, clinical manifestation, laboratory test, treatment-related, and healthcare utilization characteristics between children aged 6 months to 6 years with community-acquired influenza who required referral or hospitalization and those who did not.

Variables	Total	Non-referral or hospitalization	Referral or hospitalization	Statistic	*P*
	(*n* = 388)	(*n* = 339)	(*n* = 49)		
Age, M (Q₁, Q₃)	59.00 (42.00, 71.00)	59.00 (43.00, 72.00)	59.00 (31.00, 68.00)	Z = −1.09	0.276
Temperature, M (Q₁, Q₃)	39.00 (38.50, 39.30)	39.00 (38.50, 39.20)	39.20 (39.00, 39.90)	Z = −4.22	<.001
Time Reducing Fever, M (Q₁, Q₃)	2.00 (1.00, 3.00)	2.00 (1.00, 3.00)	3.00 (2.00, 4.00)	Z = −4.58	<.001
Number Of Visits, M (Q₁, Q₃)	1.00 (1.00, 2.00)	1.00 (1.00, 2.00)	2.00 (2.00, 3.00)	Z = −7.85	<.001
Outpatient expenses, M (Q₁, Q₃)	320.59 (260.00, 420.12)	305.41 (253.75, 399.50)	503.89 (377.63, 600.00)	Z = −6.87	<.001
Wbc, M (Q₁, Q₃)	8.29 (6.35, 10.49)	8.17 (6.33, 10.45)	8.68 (6.77, 10.94)	Z = −0.74	0.457
Ne Per, M (Q₁, Q₃)	65.20 (53.63, 74.00)	65.40 (54.25, 74.15)	63.00 (53.20, 70.50)	Z = −0.77	0.441
Lym Per, M (Q₁, Q₃)	22.80 (14.95, 32.23)	22.50 (14.70, 32.25)	25.60 (16.30, 31.60)	Z = −0.83	0.409
Ne, M (Q₁, Q₃)	5.19 (3.61, 7.46)	5.17 (3.60, 7.41)	5.20 (3.69, 7.45)	Z = −0.15	0.878
Lym, M (Q₁, Q₃)	1.69 (1.25, 2.43)	1.70 (1.25, 2.42)	1.67 (1.37, 2.73)	Z = −0.62	0.533
Hb, M (Q₁, Q₃)	129.00 (123.00, 134.00)	128.00 (122.00, 134.00)	129.00 (123.00, 134.00)	Z = −0.47	0.637
Plt, M (Q₁, Q₃)	214.00 (178.00, 252.00)	214.00 (178.00, 252.00)	212.00 (186.00, 260.00)	Z = −0.69	0.489
Sex, *n* (%)				*χ*^2^ = 0.03	0.870
Female	170 (43.81)	148 (43.66)	22 (44.90)		
Male	218 (56.19)	191 (56.34)	27 (55.10)		
School, *n* (%)				*χ*^2^ = 0.79	0.375
No	84 (21.65)	71 (20.94)	13 (26.53)		
Yes	304 (78.35)	268 (79.06)	36 (73.47)		
Diagnosis, *n* (%)				–	0.171
Influenza A	308 (79.38)	264 (77.88)	44 (89.80)		
Influenza B	77 (19.85)	72 (21.24)	5 (10.20)		
Other types	3 (0.77)	3 (0.88)	0 (0.00)		
Influenza vaccine, *n* (%)				*χ*^2^ = 29.45	<.001
No	192 (49.48)	150 (44.25)	42 (85.71)		
Yes	196 (50.52)	189 (55.75)	7 (14.29)		
Anti Influenza Drugs, *n* (%)				–	<.001
No	5 (1.29)	0 (0.00)	5 (10.20)		
Yes	383 (98.71)	339 (100.00)	44 (89.80)		
Antibiotic, *n* (%)				*χ*^2^ = 42.35	<.001
No	348 (89.69)	317 (93.51)	31 (63.27)		
Yes	40 (10.31)	22 (6.49)	18 (36.73)		
Cough, *n* (%)				*χ*^2^ = 10.16	0.001
No	143 (36.86)	135 (39.82)	8 (16.33)		
Yes	245 (63.14)	204 (60.18)	41 (83.67)		
Sal Congestion, *n* (%)				*χ*^2^ = 1.58	0.208
No	230 (59.28)	205 (60.47)	25 (51.02)		
Yes	158 (40.72)	134 (39.53)	24 (48.98)		
Runny Nose, *n* (%)				*χ*^2^ = 5.12	0.024
No	169 (43.56)	155 (45.72)	14 (28.57)		
Yes	219 (56.44)	184 (54.28)	35 (71.43)		
Sore Throat, *n* (%)				*χ*^2^ = 37.92	<.001
No	333 (85.82)	305 (89.97)	28 (57.14)		
Yes	55 (14.18)	34 (10.03)	21 (42.86)		
Muscle Soreness, *n* (%)				*χ*^2^ = 87.83	<.001
No	344 (88.66)	320 (94.40)	24 (48.98)		
Yes	44 (11.34)	19 (5.60)	25 (51.02)		
Digestive symptoms, *n* (%)				*χ*^2^ = 49.61	<.001
No	329 (84.79)	304 (89.68)	25 (51.02)		
Yes	59 (15.21)	35 (10.32)	24 (48.98)		
Crp, *n* (%)				*χ*^2^ = 33.87	<.001
No	325 (83.76)	298 (87.91)	27 (55.10)		
Yes	63 (16.24)	41 (12.09)	22 (44.90)		

M, median; Q1, first quartile; Q3, third quartile; Wbc, white blood cell count; Ne Per, neutrophil percentage; Lym Per, lymphocyte percentage; Ne, absolute neutrophil count; Lym, absolute lymphocyte count; Hb, hemoglobin; Plt, platelet count; CRP, C-reactive protein.

Children in the referral or hospitalization group exhibited more severe clinical symptoms (high fever, multi-system symptoms), more complex healthcare-seeking processes (multiple clinic visits, high outpatient costs), and higher inflammatory marker levels, while having lower vaccination rates and higher antibiotic usage rates, suggesting that these factors may be closely associated with the need for referral or hospitalization treatment in children with influenza.

### 3.2 Multivariable analysis of risk factors

Univariate analysis showed that influenza vaccination was a protective factor for referral or hospitalization in community pediatric influenza patients children with influenza compared to non-vaccination (OR = 0.13, 95%CI: 0.06 to −0.30, *P* < 0.001). Antibiotic use (OR = 8.37, 95%CI: 4.06 to −17.26, *P* < 0.001), cough symptoms (OR = 3.39, 95%CI: 1.54 to −7.46, *P* = 0.002), runny nose (OR = 2.11, 95%CI: 1.09 to −4.06, *P* = 0.026), sore throat (OR = 6.73, 95%CI: 3.45 to −13.12, *P* < 0.001), muscle aches (OR = 17.54, 95%CI: 8.48 to −36.28, *P* < 0.001), gastrointestinal symptoms (OR = 8.34, 95%CI: 4.31 to −16.14, *P* < 0.001), elevated C-reactive protein (OR = 5.92, 95%CI: 3.09 to −11.35, *P* < 0.001), elevated body temperature (OR = 3.59, 95%CI: 2.06 to −6.24, *P* < 0.001), prolonged fever clearance time (OR = 1.66, 95%CI: 1.35 to −2.04, *P* < 0.001), increased number of clinic visits (OR = 3.18, 95%CI: 2.24 to −4.52, *P* < 0.001), and increased outpatient costs (OR = 1.01, 95%CI: 1.01 to −1.01, *P* < 0.001) were all significantly associated with the risk of referral or hospitalization in community pediatric influenza patients children with influenza.

Multivariate Multivariable logistic regression analysis showed that non-vaccination against influenza (OR = 0.27, 95%CI: 0.08 to −0.94, *P* = 0.040), antibiotic use (OR = 3.52, 95%CI: 1.03 to −12.09, *P* = 0.045), sore throat (OR = 6.44, 95%CI: 2.08 to −19.88, *P* = 0.001), muscle aches (OR = 10.89, 95%CI: 3.45 to −34.33, *P* < 0.001), gastrointestinal symptoms (OR = 8.02, 95%CI: 2.62 to −24.60, *P* < 0.001), elevated C-reactive protein (OR = 4.59, 95%CI: 1.41 to −14.94, *P* = 0.011), and increased number of clinic visits (OR = 2.02, 95%CI: 1.14 to −3.58, *P* = 0.017) were independent risk factors for referral or hospitalization in community pediatric influenza patients children with influenza (*P* < 0.05) (Table 2).

**Table 2:** Univariate and multivariable logistic regression analyses of independent risk factors associated with referral or hospitalization in children aged 6 months to 6 years with community-acquired influenza. This table presents the regression coefficient, odds ratio (OR), 95% confidence interval (95%CI), and *P* value of each included variable.

Variables	Univariate			Multivariable		
	*β*	*P*	OR (95%CI)	β	*P*	OR (95%CI)
Sex						
Female			1.00 (Reference)			
Male	−0.05	0.870	0.95 (0.52–1.74)			
School						
No			1.00 (Reference)			
Yes	−0.31	0.376	0.73 (0.37–1.46)			
Diagnosis						
Influenza A			1.00 (Reference)			
Influenza B	−0.88	0.074	0.42 (0.16–1.09)			
Other types	−13.77	0.987	0.00 (0.00–Inf)			
Influenza vaccine						
No			1.00 (Reference)			1.00 (Reference)
Yes	−2.02	<.001	0.13 (0.06–0.30)	−1.32	0.040	0.27 (0.08 0.94)
Anti Influenza Drugs						
No			1.00 (Reference)			
Yes	−18.61	0.986	0.00 (0.00–Inf)			
Antibiotic						
No			1.00 (Reference)			1.00 (Reference)
Yes	2.12	<.001	8.37 (4.06–17.26)	1.26	0.045	3.52 (1.03–12.09)
Cough						
No			1.00 (Reference)			
Yes	1.22	0.002	3.39 (1.54–7.46)			
Sal Congestion						
No			1.00 (Reference)			
Yes	0.38	0.210	1.47 (0.8–2.68)			
Runny Nose						
No			1.00 (Reference)			
Yes	0.74	0.026	2.11 (1.09–4.06)			
Sore Throat						
No			1.00 (Reference)			1.00 (Reference)
Yes	1.91	<.001	6.73 (3.45–13.12)	1.86	0.001	6.44 (2.08–19.88)
Muscle Soreness						
No			1.00 (Reference)			1.00 (Reference)
Yes	2.86	<.001	17.54 (8.48–36.28)	2.39	<.001	10.89 (3.45–34.33)
Digestive symptoms						
No			1.00 (Reference)			1.00 (Reference)
Yes	2.12	<.001	8.34 (4.31–16.14)	2.08	<.001	8.02 (2.62–24.60)
Crp						
No			1.00 (Reference)			1.00 (Reference)
Yes	1.78	<.001	5.92 (3.09–11.35)	1.52	0.011	4.59 (1.41–14.94)
Age	−0.01	0.157	0.99 (0.98–1.00)			
Temperature	1.28	<.001	3.59 (2.06–6.24)			
Time Reducing Fever	0.51	<.001	1.66 (1.35–2.04)			
Number Of Visits	1.16	<.001	3.18 (2.24–4.52)	0.70	0.017	2.02 (1.14–3.58)
Outpatient expenses	0.01	<.001	1.01 (1.01–1.01)			
Wbc	0.01	0.884	1.01 (0.92–1.10)			
Ne Per	−0.00	0.753	1.00 (0.98–1.02)			
Lym Per	0.00	0.714	1.00 (0.98–1.03)			
Ne	−0.01	0.905	0.99 (0.89–1.10)			
Lym	0.01	0.907	1.01 (0.80–1.28)			
Hb	−0.00	0.963	1.00 (0.97–1.03)			
Plt	0.00	0.391	1.00 (1.00–1.01)			

β, regression coefficient; OR, odds ratio; 95%CI, 95% confidence interval; Wbc, white blood cell count; Ne Per, neutrophil percentage; Lym Per, lymphocyte percentage; Ne, absolute neutrophil count; Lym, absolute lymphocyte count; Hb, hemoglobin; Plt, platelet count; CRP, C-reactive protein.

### 3.3 Construction of a nomogram

**Figure 2:**
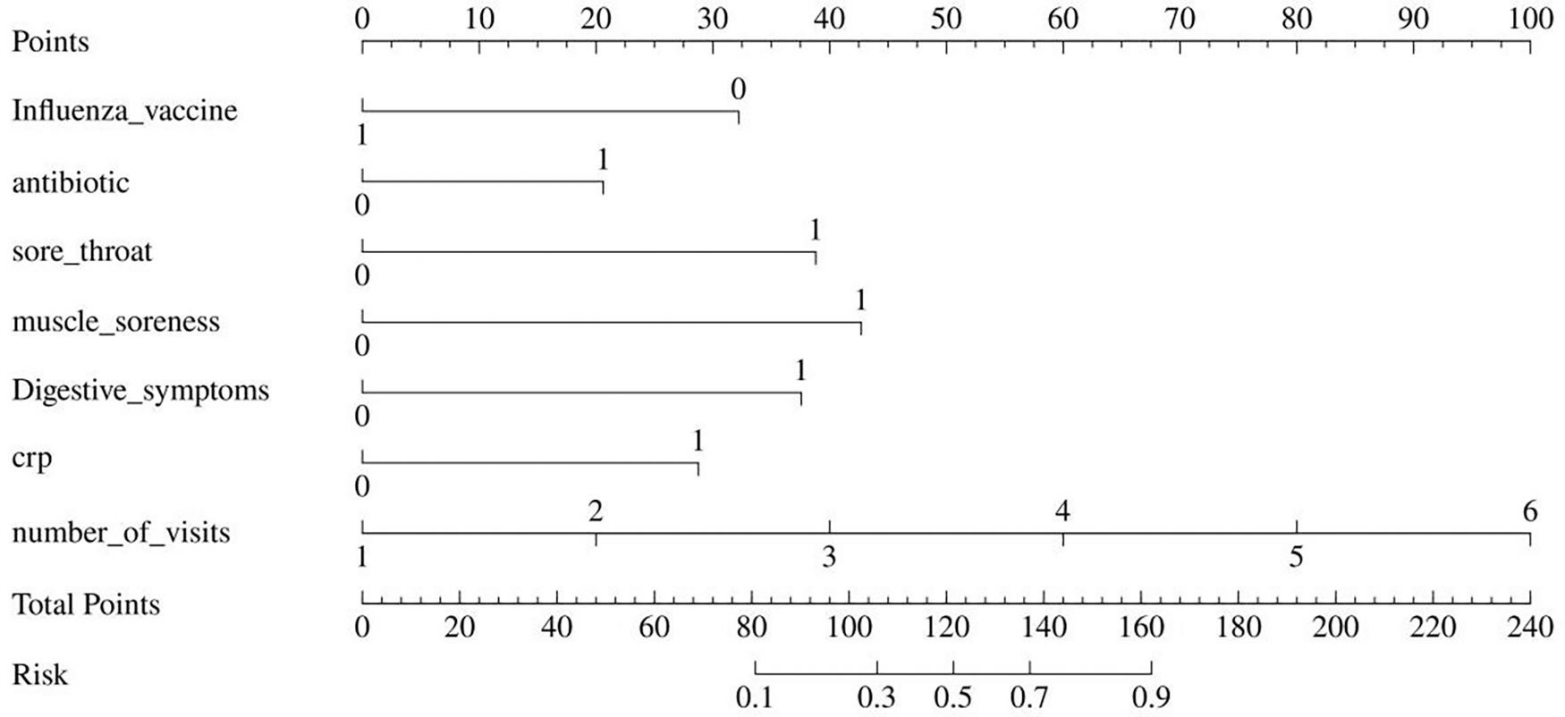
Nomogram for predicting the risk of referral or hospitalization in children aged 6 months to 6 years with community-acquired influenza. This nomogram was developed based on 7 independent risk factors identified by multivariable logistic regression analysis, including lack of influenza vaccination, antibiotic usage, sore throat, myalgia, gastrointestinal symptoms, elevated C-reactive protein, and increased frequency of medical visits.

Based on the 7 independent risk factors identified through multivariable logistic regression analysis (non-vaccination against influenza, antibiotic use, sore throat, muscle aches, gastrointestinal symptoms, elevated C-reactive protein, and increased number of clinic visits), a nomogram prediction model was constructed using the “rms” package in R language (Figure 2). This nomogram enabled individualized risk assessment through scoring: each predictor corresponded to a score on the horizontal “Points” scale, the scores were summed to obtain the “Total Points”, which then corresponded to the vertical “Risk” axis to read the predicted probability of referral or hospitalization for children. To ensure clinical practicability and computational precision, we added Supplementary Material Tables S3 and S4 to provide exact point allocations for all predictor variable categories.

### 3.4 ROC and AUC of the model

**Figure 3:**
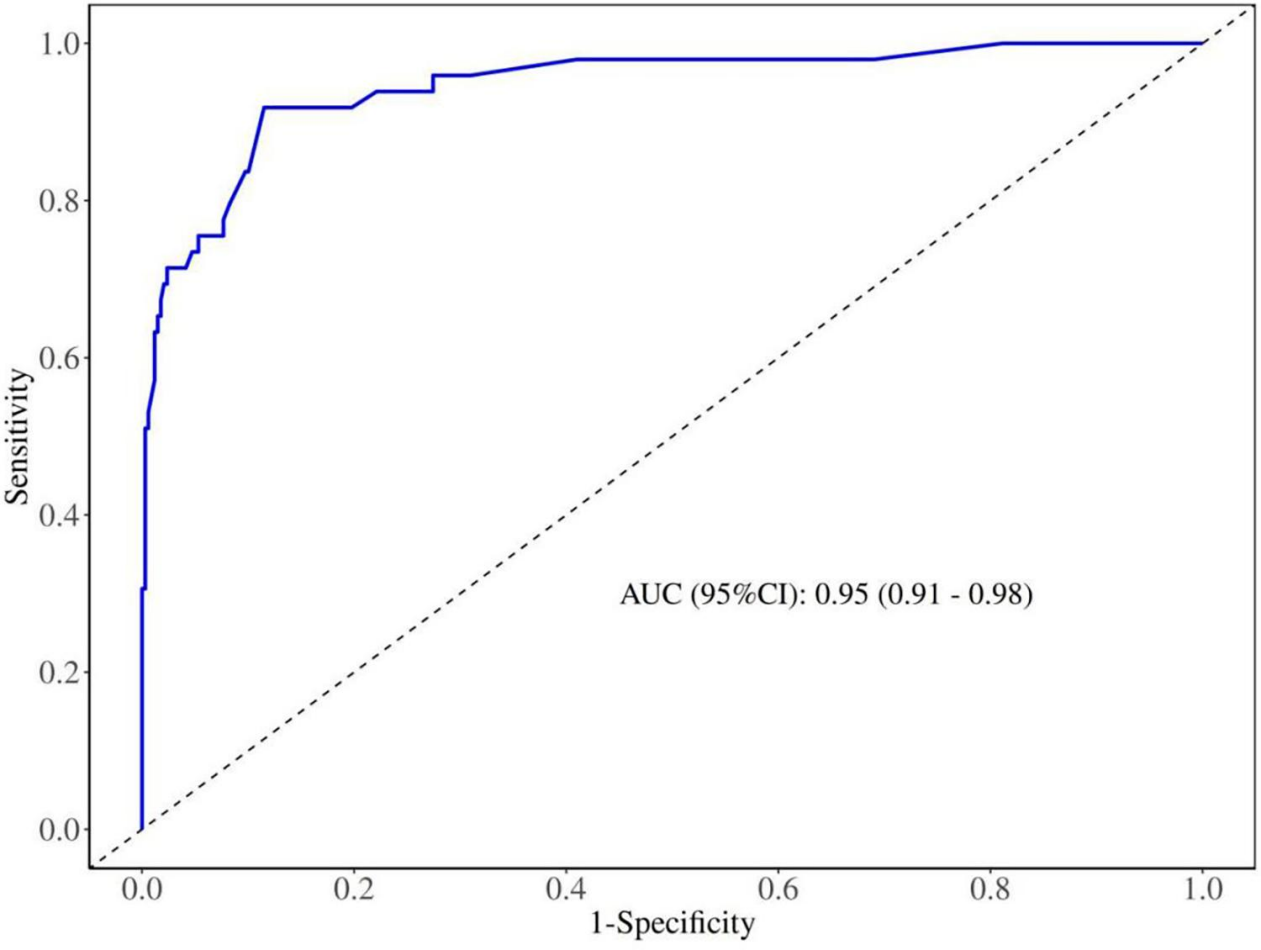
Receiver operating characteristic (ROC) curve of the primary nomogram model for predicting referral or hospitalization risk in children aged 6 months to 6 years with community-acquired influenza. The curve was used to evaluate the discriminative ability of the prediction model.

The discriminative ability of the nomogram model was evaluated using AUC values. As shown in Figure 3 and Table 3, the nomogram model constructed based on 7 independent risk factors demonstrated excellent discriminative ability, with an area under the ROC curve (AUC) of 0.95 (95% CI: 0.92–0.98). Using a cutoff value of 0.144, the model achieved an accuracy of 0.88 (95% CI: 0.85–0.91), sensitivity of 0.92 (95% CI: 0.84–1.00), and specificity of 0.88 (95% CI: 0.84–0.91). These data indicated that the model had good discriminative ability and predictive value, capable of correctly identifying whether community influenza children require referral or hospitalization. Additionally, the variance inflation factor (VIF) for each predictor variable was less than 2, indicating no serious multicollinearity issues. (VIF: vaccination status: 1.073, antibiotic use: 1.146, sore throat: 1.058, muscle aches: 1.139, gastrointestinal symptoms: 1.086, C-reactive protein: 1.105, number of visits: 1.096).

**Table 3:** Predictive performance metrics of the primary nomogram model for predicting referral or hospitalization risk in children aged 6 months to 6 years with community-acquired influenza. This table presents the area under the ROC curve (AUC), accuracy, sensitivity, specificity, and optimal cutoff value of the model.

AUC (95%CI)	Accuracy (95%CI)	Sensitivity (95%CI)	Specificity (95%CI)	cut off
0.95 (0.92–0.98)	0.88 (0.85–0.91)	0.92 (0.84–1.00)	0.88 (0.84–0.91)	0.144

AUC, area under the receiver operating characteristic curve; 95%CI, 95% confidence interval.

### 3.5 Calibration curve of the model

**Figure 4:**
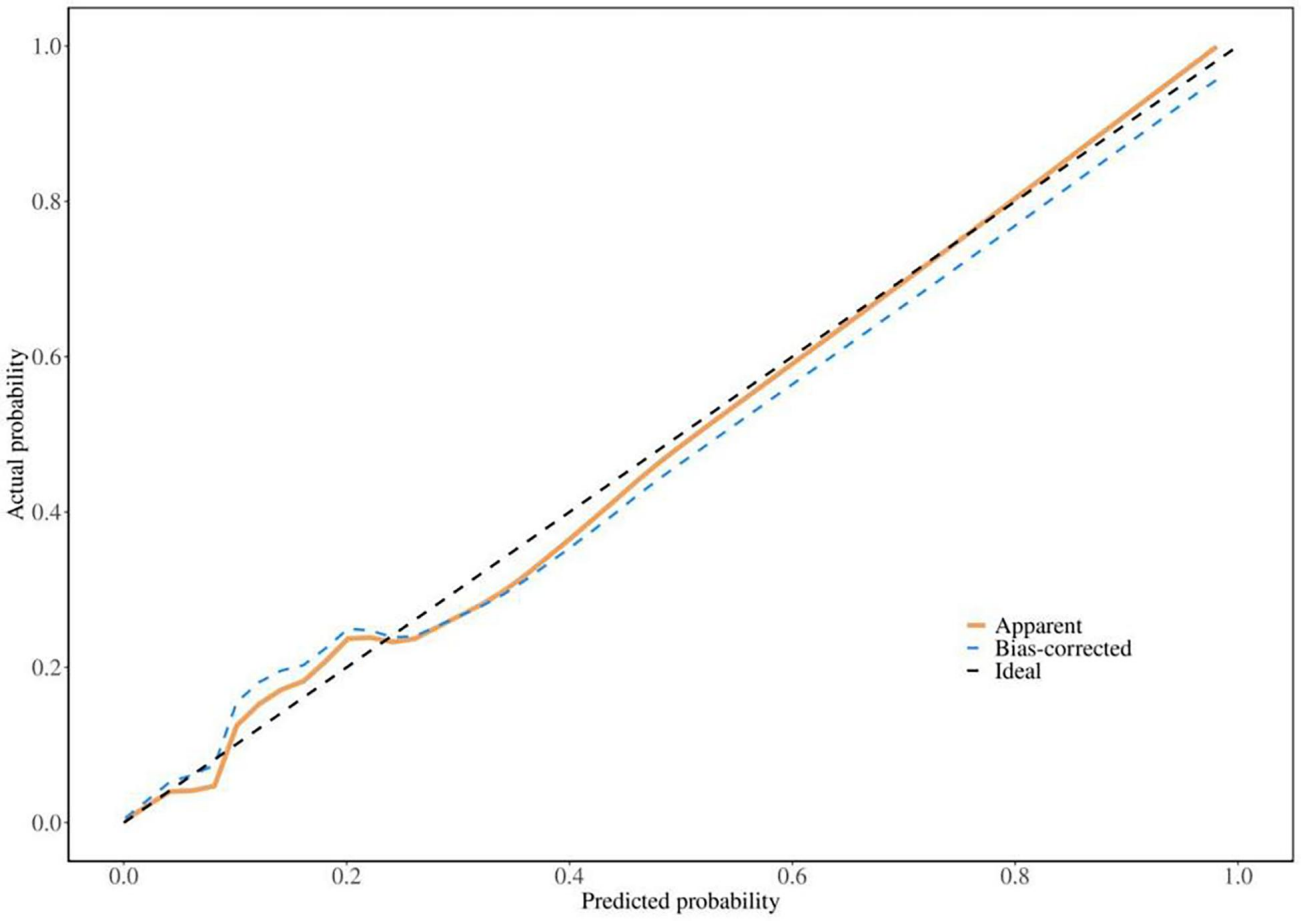
Calibration curve of the model calibration curve of the primary nomogram model for predicting referral or hospitalization risk in children aged 6 months to 6 years with community-acquired influenza. The curve was used to assess the consistency between the model-predicted probability and the actual observed probability of the primary endpoint.

Calibration curves and the Hosmer-Lemeshow goodness-of-fit test were used to evaluate the consistency between actual outcomes and predicted probabilities. Calibration curves derived from the multivariate logistic regression model were presented in Figure 4 and Supplementary Figure S1, demonstrating good agreement between observed and predicted values with favorable consistency. The Hosmer-Lemeshow goodness-of-fit test yielded a *P*-value greater than 0.05 (*χ*^2^ = 2.5576, df = 6, *P* = 0.862), with a calibration slope of 1.083, indicating a good fit of the nomogram model.

### 3.6 Clinical decision curve of the model

**Figure 5:**
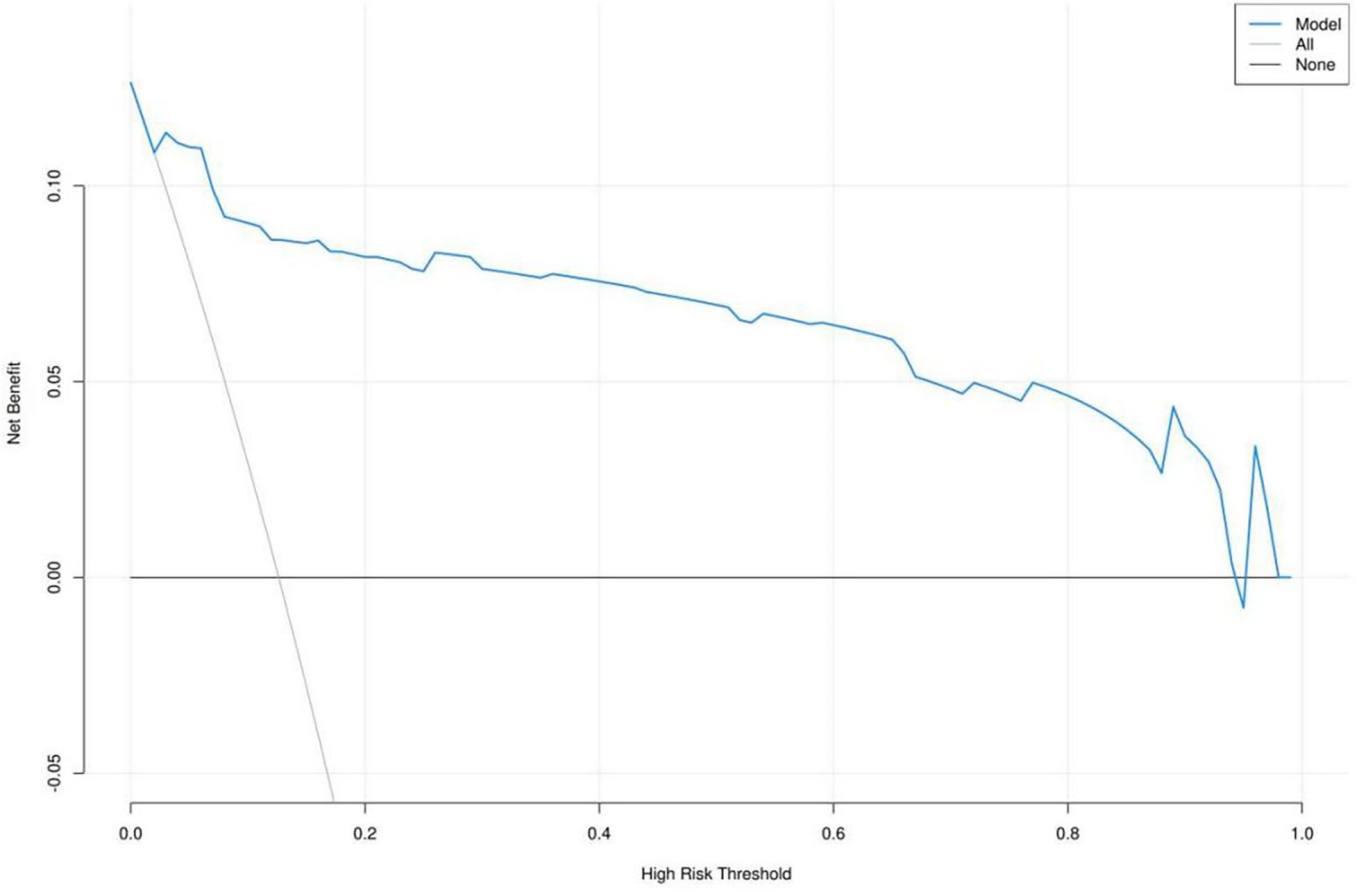
Clinical decision curve of the model decision curve analysis (DCA) curve of the primary nomogram model for predicting referral or hospitalization risk in children aged 6 months to 6 years with community-acquired influenza. The curve was used to evaluate the clinical net benefit and clinical utility of the prediction model across different threshold probabilities.

Clinical decision curves were applied to assess the clinical validity of the nomogram. According to the DCA, interventions guided by the predictive model demonstrated superior performance, except for a small range of low preference thresholds (Figure 5). The results indicated that the nomogram model generated significant net benefit, meaning our model had robust predictive accuracy and clinical efficacy in predicting the risk of referral or hospitalization for children with influenza in community settings.

### 3.7 Variable importance ranking based on SHAP

**Figure 6:**
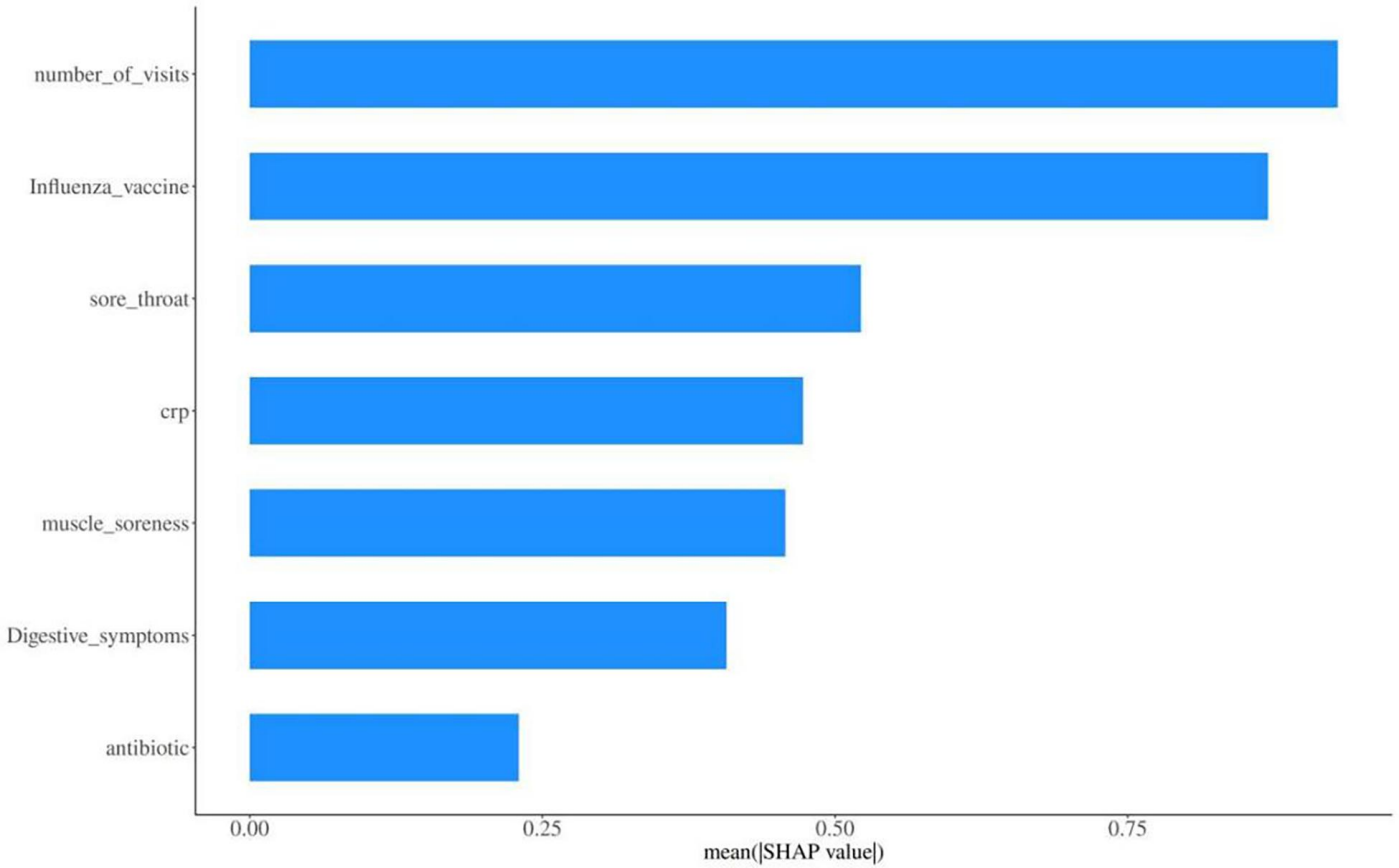
Variable importance ranking based on SHAP Variable importance ranking based on SHapley additive exPlanations (SHAP) method for the primary nomogram model. This ranking quantifies the relative contribution of each independent risk factor to the prediction of referral or hospitalization risk in children aged 6 months to 6 years with community-acquired influenza.

To further evaluate the relative importance of each predictor variable on the model's prediction results, this study employed the SHAP (SHapley Additive exPlanations) method to conduct interpretability analysis of the constructed predictive model. As shown in the SHAP variable importance ranking results in Figure 6, the number of medical visits was the most important predictor affecting the risk of referral or hospitalization for community children with influenza aged 6 months to 6 years, followed by influenza vaccination status. Sore throat and C-reactive protein had similar importance, ranking 3rd and 4th respectively. Muscle aches and gastrointestinal symptoms showed relatively high importance, while antibiotic use had relatively low importance. The SHAP analysis results were highly consistent with the independent risk factors identified by multivariable logistic regression analysis, further validating the reliability of the model.

### 3.8 Bootstrap internal validation

**Figure 7:**
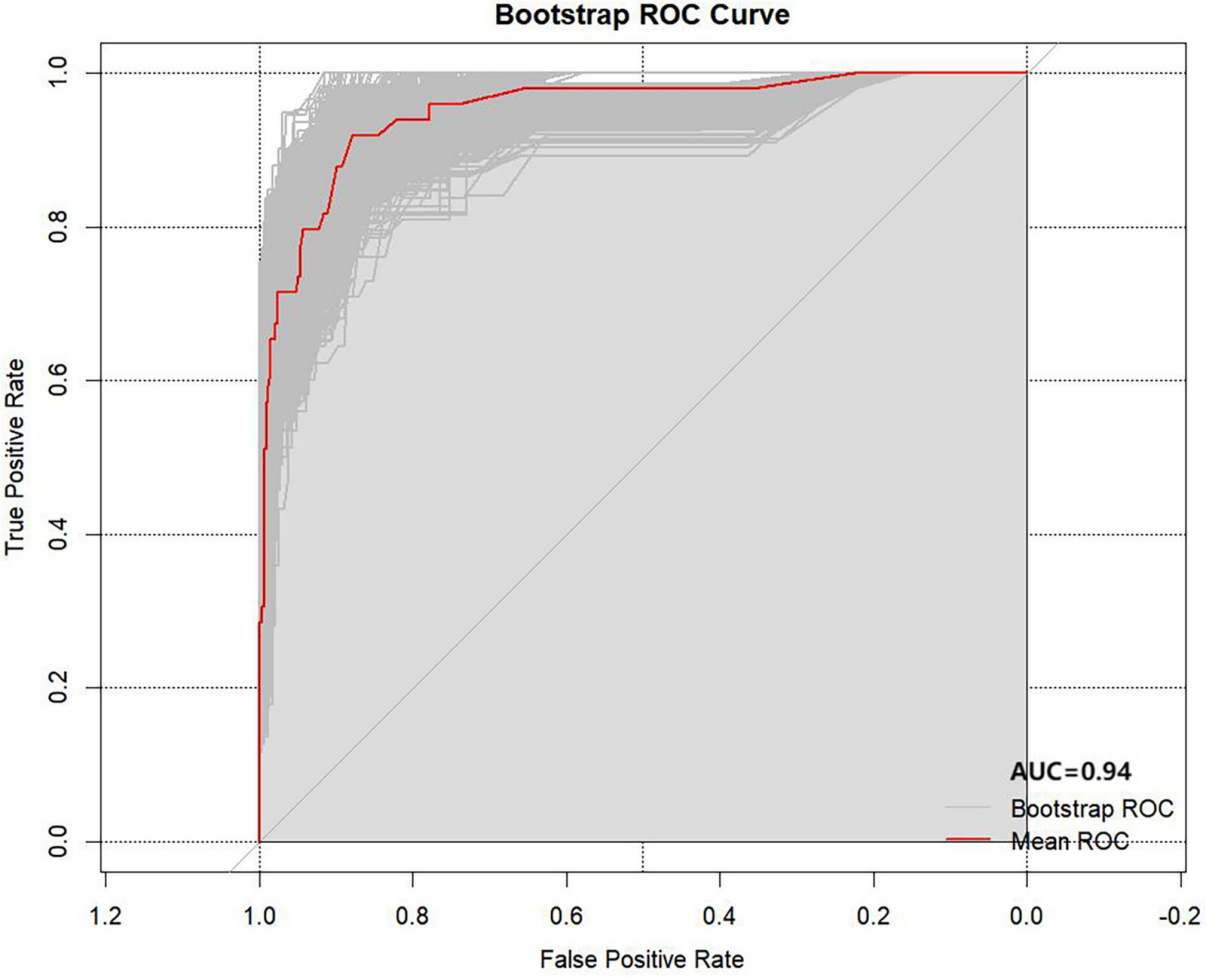
Receiver operating characteristic (ROC) curve of the primary nomogram model after 1,000 iterations of Bootstrap internal validation. The curve was used to verify the stability and discriminative ability of the model for predicting referral or hospitalization risk in children aged 6 months to 6 years with community-acquired influenza.

The Bootstrap analysis results showed that through 1,000 resampling procedures, as shown in Figure 7, the final model achieved an area under the ROC curve of 0.94 (95% CI: 0.91–0.97), with sensitivity = 0.97, specificity = 0.64, and accuracy = 0.93. This further validated the robustness and stability of the model. Calibration curves and the calibration slopes indicated a good fit for the Bootstrap internal validation (Supplementary Figure S2).

### 3.9 Sensitivity analysis and novel model performance

To evaluate the robustness of the primary model, a novel nomogram model was constructed by excluding antibiotic use and frequency of medical visits (variables potentially influenced by physician behavior). The novel model included 5 independent baseline risk factors: lack of influenza vaccination, sore throat, myalgia, gastrointestinal symptoms, and elevated CRP (Supplementary Material Figure S3). The novel model exhibited good discriminative ability, with an AUC of 0.92 (95% CI: 0.88–0.96), accuracy of 0.82, sensitivity of 0.94, and specificity of 0.81 at the optimal cutoff value of 0.051 (Supplementary Material Figure S4, Table S5). Calibration curves showed excellent consistency between predicted and observed probabilities (HL test: *χ*^2^ = 4.2536, df = 3, *P* = 0.2354; calibration slope = 1.252) (Supplementary Material Figures S5, S8). DCA demonstrated that the novel model still yielded significant clinical net benefit across most preference thresholds, indicating its reliable clinical utility (Supplementary Material Figure S6). The optimized SHAP importance ranking plot (Supplementary Material Figure S7) clearly quantifies the contribution of each independent risk factor to the prediction outcome. Bootstrap internal validation with 1,000 iterations confirmed the stability of the novel model, with a validated AUC of 0.92 (95% CI: 0.88–0.96), sensitivity of 0.97, specificity of 0.55, and accuracy of 0.92 (Supplementary Material Figure S9). Calibration curves and the calibration slopes indicated a good fit for the Bootstrap internal validation (Supplementary Material Figure S10). Comparison of the primary and novel models showed that the discriminative ability of the novel model slightly decreased but remained at a high level, suggesting that the primary model had good robustness even after excluding variables with potential bias.

## 4 Discussion

Based on clinical data from 388 community children with influenza, this study successfully constructed a nomogram model for predicting the risk of referral or hospitalization. The model integrated 7 clinically accessible predictive factors and demonstrated good discrimination, calibration, and clinical utility, providing an effective tool for clinical decision-making in primary healthcare institutions. The study results showed that lack of influenza vaccination, antibiotic use, sore throat, muscle aches, gastrointestinal symptoms, elevated C-reactive protein, and increased number of medical visits were independent risk factors for referral or hospitalization in children with influenza. These findings were generally consistent with previous research results.

Influenza vaccination can significantly reduce the incidence and severity of influenza, making it an effective preventive measure. This study found that lack of influenza vaccination was an independent risk factor for referral or hospitalization among community children with influenza aged 6 months to 6 years, which was consistent with multiple domestic and international studies. Fu et al. (11) conducted a matched case–control study in Guangzhou during the 2010–2011 and 2011–2012 influenza seasons and reported that, among children aged 6–59 months, the trivalent inactivated influenza vaccine provided moderate protection, with vaccine effectiveness (VE) of 73.2% (95% CI: 52.2–85.0) in 2010–2011 and 52.9% (95% CI: 42.1–61.7) in 2011–2012; protection tended to be higher in children aged 36–59 months than in younger children. Using routine surveillance data from five consecutive seasons in Taiwan, Su et al. (12) further showed that influenza vaccination in children aged 6–59 months was effective overall, with a pooled VE of 62% (95% CI: 48–83), and age-specific VEs of 51% (95% CI: 23–68) in children 6–23 months and 75% (95% CI: 60–84) in children 24–59 months. These studies all indicate that influenza vaccines can provide moderate to good protective effects for children. International research also supports our findings. A multicenter study covering 9 Latin American countries showed that fully vaccinated children under 5 years of age had a 47% protective effect against influenza-associated severe acute respiratory infections (13). Wang et al. (14)'s global systematic review found that influenza virus infections caused approximately 109.5 million cases in children under 5 years of age, while vaccination could significantly reduce the risk of hospitalization.

The occurrence of systemic symptoms such as muscle aches and gastrointestinal symptoms often indicates severe illness and possible viremia or multi-system involvement (15). These symptoms may be related to the release of pro-inflammatory cytokines such as IL-6 and TNF-α induced by influenza virus infection. These cytokines trigger systemic inflammatory responses, indicating viremia or multi-system involvement, which is consistent with previous research findings that “systemic symptoms are warning signs of severe influenza (16).” Although sore throat is a common symptom of influenza, its incidence rate is significantly higher in children who require referral or hospitalization, which may be related to more severe inflammatory responses (17). C-reactive protein, as an acute-phase reactant protein, reflects the degree of inflammatory response in the body when elevated, and can serve as an objective indicator for assessing the severity of illness (18).

It is worth noting that antibiotic use and increased number of medical visits are also independent risk factors for referral or hospitalization in children with influenza. Antibiotic use as an independent risk factor does not mean that antibiotics directly cause disease deterioration, but rather reflects the primary care physicians' prediction of influenza complicated by bacterial infection, indirectly indicating disease complexity. This suggests that clinical practice needs to strictly distinguish between viral and bacterial infections to avoid blind antibiotic use (19). Multiple medical visits often indicate persistent or worsening symptoms and poor disease control (20). These findings suggest that clinicians should maintain high vigilance when encountering such situations and promptly assess changes in the patient's condition. To further address the potential bias of these two variables, we performed a sensitivity analysis by constructing a novel baseline-only model excluding antibiotic use and frequency of medical visits. The results showed that the novel model still maintained excellent predictive performance, indicating that the primary model's conclusion was robust and the core risk factors had stable predictive value for referral or hospitalization risk. This finding suggests that although antibiotic use and medical visits are affected by clinical decision-making, they do not alter the identification of the core baseline risk factors for severe influenza in children, which further supports the clinical applicability of the primary model.

The nomogram model constructed in this study has the following advantages: (1) It filled the gap in risk stratification tools for referral or hospitalization of children with influenza in primary healthcare institutions, providing scientific evidence for clinical decision-making; (2) All predictive factors were common and easily obtainable clinical indicators, facilitating widespread application in resource-limited primary healthcare institutions; (3) The nomogram format was intuitive and easy to understand, allowing clinicians to quickly calculate individualized risk probabilities and improve decision-making efficiency; (4) The model's discriminative ability, calibration, and clinical utility had been validated through multiple methods including Bootstrap internal validation, ROC curve analysis, calibration curves, and DCA analysis.

However, this study also had certain limitations. First, this was a single-center prospective cohort study with a relatively limited sample size and lack of external validation; the extrapolability and generalizability of the model require further validation through multicenter, large-sample studies. Second, due to objective constraints, indicators such as bacterial culture, viral load, and chest imaging were not included, which may had missed some severe illness warning signals. Third, recall bias may exist regarding vaccination history and past medical history in some children. Fourth, certain variables such as antibiotic use may reflect clinicians' suspicion of bacterial co-infection or disease severity, rather than representing independent risk factors. Similarly, multiple outpatient visits may indicate persistent symptoms, but could also be indicative of healthcare practice patterns or parental anxiety. These factors may introduce potential reverse causality and confounding by indication. Finally, this study primarily focused on outpatient children, which may introduce selection bias regarding children with mild self-limiting illness or severe cases requiring direct hospitalization. Future research should focus on multicenter, large-sample study cohorts for further validation.

## 5 Conclusions

The nomogram prediction model constructed based on easily obtainable clinical indicators had good predictive performance for the risk of referral or hospitalization in community children with influenza. It provided an effective tool for clinical decision-making in primary healthcare institutions, helping to achieve precision medicine and rational allocation of medical resources. Future multicenter prospective studies are needed to further validate and optimize this model.

## Data Availability

The data analyzed in this study is subject to the following licenses/restrictions: Due to patient privacy and ethical restrictions, the datasets generated and/or analyzed during the current study are not publicly available but are available from the corresponding author on reasonable request and with permission of the institutional ethics committee. Requests to access these datasets should be directed to Xueying Meng, 2545517835@qq.com.
