# Supplementary material for: Development and internal validation of a nomogram for predicting referral or hospitalization risk in children with community-acquired influenza
Source: Front Pediatr. 2026 Apr 10;14:1779925. doi: 10.3389/fped.2026.1779925 (PMC13106406; doi:10.3389/fped.2026.1779925)
Supplement: Supplementary file 1 [file Supplementaryfile1.docx]

**Supplementary Material Table 1** Missing rate (percentage) of each variable included in the primary nomogram model developed to predict the risk of referral or hospitalization in children aged 6 months to 6 years with community-acquired influenza.

| **Index** | **Missingness rate (percentage)** |
| --- | --- |
| Hospitalization | 0 |
| Sex | 0 |
| School | 0 |
| Diagnosis | 0 |
| Influenza_vaccine | 3.608247 |
| Anti_influenza_drugs | 4.639175 |
| Antibiotic | 2.577320 |
| Cough | 0 |
| Sal_congestion | 0 |
| Runny_nose | 0 |
| Sore_throat | 0 |
| Muscle_soreness | 0 |
| Digestive_symptoms | 0 |
| Crp | 2.319588 |
| Age | 0 |
| Temperature | 0 |
| Time_reducing_fever | 1.546392 |
| Number_of_visits | 3.350515 |
| Outpatient_expenses | 4.123711 |
| Wbc | 6.701031 |
| Ne_per | 6.185567 |
| Lym_per | 4.639175 |
| Ne | 6.443299 |
| Lym | 4.123711 |
| Hb | 3.865979 |
| Plt | 5.670103 |

Abbreviations: Wbc, white blood cell count; Ne Per, neutrophil percentage; Lym Per, lymphocyte percentage; Ne, absolute neutrophil count; Lym, absolute lymphocyte count; Hb, hemoglobin; Plt, platelet count; CRP, C-reactive protein.

**Supplementary Material Table 2** Differential analysis between the original dataset and the dataset after multiple imputation by chained equations (MICE), for variables included in the prediction model of referral or hospitalization risk in children aged 6 months to 6 years with community-acquired influenza.

| **Variables** | **Original dataset** | **Imputed dataset** | **Statistic** | **P** |
| --- | --- | --- | --- | --- |
| Age, M (Q₁, Q₃) | 59.00 (42.00, 71.00) | 59.00 (42.00, 71.00) | Z=0.00 | 1.000 |
| Temperature, M (Q₁, Q₃) | 39.00 (38.50, 39.30) | 39.00 (38.50, 39.30) | Z=0.00 | 1.000 |
| Time Reducing Fever, M (Q₁, Q₃) | 2.00 (1.00, 3.00) | 2.00 (1.00, 3.00) | Z=-0.01 | 0.990 |
| Number Of Visits, M (Q₁, Q₃) | 1.00 (1.00, 2.00) | 1.00 (1.00, 2.00) | Z=-0.10 | 0.921 |
| Outpatient expenses, M (Q₁, Q₃) | 328.66 (260.00, 421.11) | 320.59 (260.00, 420.12) | Z=-0.17 | 0.866 |
| Wbc, M (Q₁, Q₃) | 8.29 (6.35, 10.36) | 8.29 (6.35, 10.49) | Z=-0.08 | 0.935 |
| Ne Per, M (Q₁, Q₃) | 65.10 (53.20, 74.12) | 65.20 (53.63, 74.00) | Z=-0.00 | 0.998 |
| Lym Per, M (Q₁, Q₃) | 22.60 (14.85, 31.58) | 22.80 (14.95, 32.23) | Z=-0.19 | 0.846 |
| Ne, M (Q₁, Q₃) | 5.15 (3.58, 7.50) | 5.19 (3.61, 7.46) | Z=-0.20 | 0.839 |
| Lym, M (Q₁, Q₃) | 1.69 (1.25, 2.42) | 1.69 (1.25, 2.43) | Z=-0.02 | 0.982 |
| Hb, M (Q₁, Q₃) | 129.00 (123.00, 134.00) | 129.00 (123.00, 134.00) | Z=-0.10 | 0.917 |
| Plt, M (Q₁, Q₃) | 213.50 (178.00, 252.00) | 214.00 (178.00, 252.00) | Z=-0.06 | 0.951 |
| Hospitalization, n(%) |  |  | χ²=0.00 | 1.000 |
| No | 339 (87.37) | 339 (87.37) |  |  |
| Yes | 49 (12.63) | 49 (12.63) |  |  |
| Sex, n(%) |  |  | χ²=0.00 | 1.000 |
| No | 170 (43.81) | 170 (43.81) |  |  |
| Yes | 218 (56.19) | 218 (56.19) |  |  |
| School, n(%) |  |  | χ²=0.00 | 1.000 |
| No | 84 (21.65) | 84 (21.65) |  |  |
| Yes | 304 (78.35) | 304 (78.35) |  |  |
| Diagnosis, n(%) |  |  | - | 1.000 |
| Influenza A | 308 (79.38) | 308 (79.38) |  |  |
| Influenza B | 77 (19.85) | 77 (19.85) |  |  |
| Other types | 3 (0.77) | 3 (0.77) |  |  |
| Influenza vaccine, n(%) |  |  | χ²=0.00 | 0.996 |
| No | 185 (49.47) | 192 (49.48) |  |  |
| Yes | 189 (50.53) | 196 (50.52) |  |  |
| Anti Influenza Drugs, n(%) |  |  | χ²=0.00 | 1.000 |
| No | 4 (1.08) | 5 (1.29) |  |  |
| Yes | 366 (98.92) | 383 (98.71) |  |  |
| Antibiotic, n(%) |  |  | χ²=0.00 | 0.997 |
| No | 339 (89.68) | 348 (89.69) |  |  |
| Yes | 39 (10.32) | 40 (10.31) |  |  |
| Cough, n(%) |  |  | χ²=0.00 | 1.000 |
| No | 143 (36.86) | 143 (36.86) |  |  |
| Yes | 245 (63.14) | 245 (63.14) |  |  |
| Sal Congestion, n(%) |  |  | χ²=0.00 | 1.000 |
| No | 230 (59.28) | 230 (59.28) |  |  |
| Yes | 158 (40.72) | 158 (40.72) |  |  |
| Runny Nose, n(%) |  |  | χ²=0.00 | 1.000 |
| No | 169 (43.56) | 169 (43.56) |  |  |
| Yes | 219 (56.44) | 219 (56.44) |  |  |
| Sore Throat, n(%) |  |  | χ²=0.00 | 1.000 |
| No | 333 (85.82) | 333 (85.82) |  |  |
| Yes | 55 (14.18) | 55 (14.18) |  |  |
| Muscle Soreness, n(%) |  |  | χ²=0.00 | 1.000 |
| No | 344 (88.66) | 344 (88.66) |  |  |
| Yes | 44 (11.34) | 44 (11.34) |  |  |
| Digestive symptoms, n(%) |  |  | χ²=0.00 | 1.000 |
| No | 329 (84.79) | 329 (84.79) |  |  |
| Yes | 59 (15.21) | 59 (15.21) |  |  |
| Crp, n(%) |  |  | χ²=0.00 | 0.964 |
| No | 317 (83.64) | 325 (83.76) |  |  |
| Yes | 62 (16.36) | 63 (16.24) |  |  |

Abbreviations: M, median; Q1, first quartile; Q3, third quartile; Wbc, white blood cell count; Ne Per, neutrophil percentage; Lym Per, lymphocyte percentage; Ne, absolute neutrophil count; Lym, absolute lymphocyte count; Hb, hemoglobin; Plt, platelet count; CRP, C-reactive protein.

**Supplementary Material Table 3** Exact point allocation for each category of the 7 independent predictive factors in the primary nomogram model for predicting referral or hospitalization risk in children aged 6 months to 6 years with community-acquired influenza.

| **Index** | **Variables** | **Points** |
| --- | --- | --- |
| Influenza_vaccine | 1 | 0 |
|  | 0 | 32 |
| Antibiotic | 1 | 20 |
|  | 0 | 0 |
| Sore_throat | 1 | 39 |
|  | 0 | 0 |
| Muscle_soreness | 1 | 42 |
|  | 0 | 0 |
| Digestive_symptoms | 1 | 37 |
|  | 0 | 0 |
| Crp | 1 | 28 |
|  | 0 | 0 |
| Number_of_visits | 1 | 0 |
|  | 2 | 18 |
|  | 3 | 38 |
|  | 4 | 58 |
|  | 5 | 78 |
|  | 6 | 100 |

Abbreviations: CRP, C-reactive protein.

**Supplementary Material Table 4** Total score of the primary nomogram model and the corresponding predicted probability interval of referral or hospitalization in children aged 6 months to 6 years with community-acquired influenza.

| **Risk Probability** | **Total score** |
| --- | --- |
| 0.1（10%） | 80 |
| 0.3（30%） | 105 |
| 0.5（50%） | 122 |
| 0.7（70%） | 136 |
| 0.9（90%） | 162 |

**Supplementary Material Table 5** Predictive performance metrics of the baseline-only nomogram model constructed in the sensitivity analysis, which was developed to predict referral or hospitalization risk in children aged 6 months to 6 years with community-acquired influenza after excluding variables with potential bias (antibiotic use and frequency of medical visits). This table presents the area under the receiver operating characteristic curve (AUC), accuracy, sensitivity, specificity, and optimal cutoff value of the model.

| **AUC (95%CI)** | **Accuracy (95%CI)** | **Sensitivity (95%CI)** | **Specificity (95%CI)** | **cut off** |
| --- | --- | --- | --- | --- |
|  |  |  |  |  |
| 0.92 (0.88-0.96) | 0.82 (0.78-0.86) | 0.94 (0.87 - 1.00) | 0.81 (0.76 - 0.85) | 0.051 |

**Supplementary Material Figure 1** Calibration curve and calibration slope of the primary nomogram model in the apparent dataset, used to assess the goodness of fit and consistency between model-predicted probability and actual observed probability of referral or hospitalization in children aged 6 months to 6 years with community-acquired influenza.


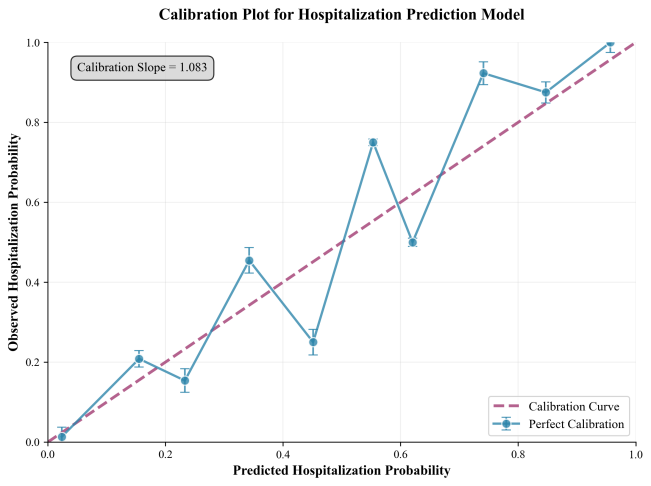


**Supplementary Material Figure 2** Calibration curves and calibration slopes of the primary nomogram model after 1000 iterations of Bootstrap internal validation, used to verify the stability and goodness of fit of the model for predicting referral or hospitalization risk in children aged 6 months to 6 years with community-acquired influenza.

**
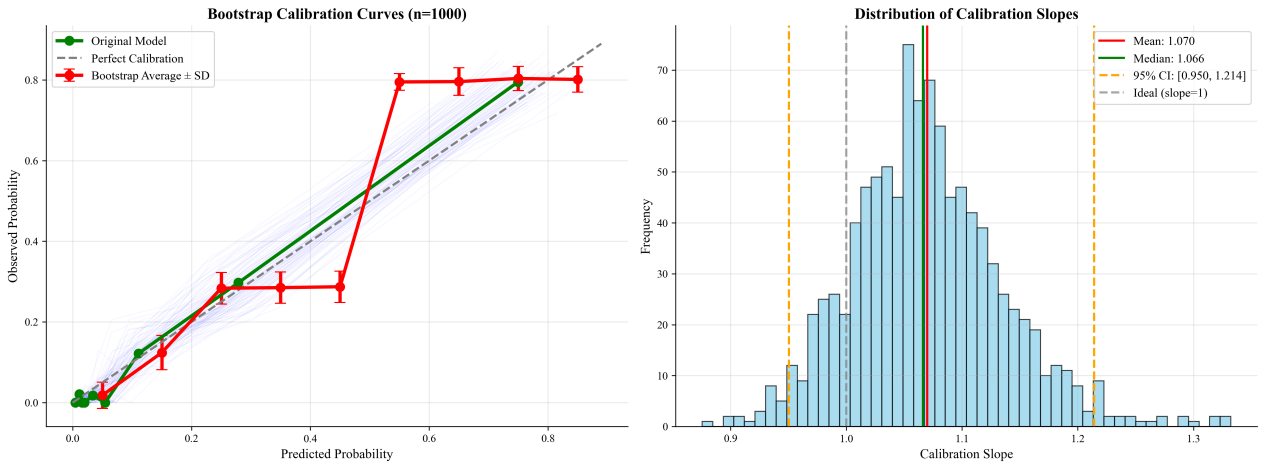
**

**Supplementary Material Figure 3** Nomogram of the baseline-only model constructed in the sensitivity analysis for predicting the risk of referral or hospitalization in children aged 6 months to 6 years with community-acquired influenza. This nomogram was developed based on 5 baseline independent risk factors, after excluding variables with potential reverse causality bias (antibiotic use and frequency of medical visits).

**
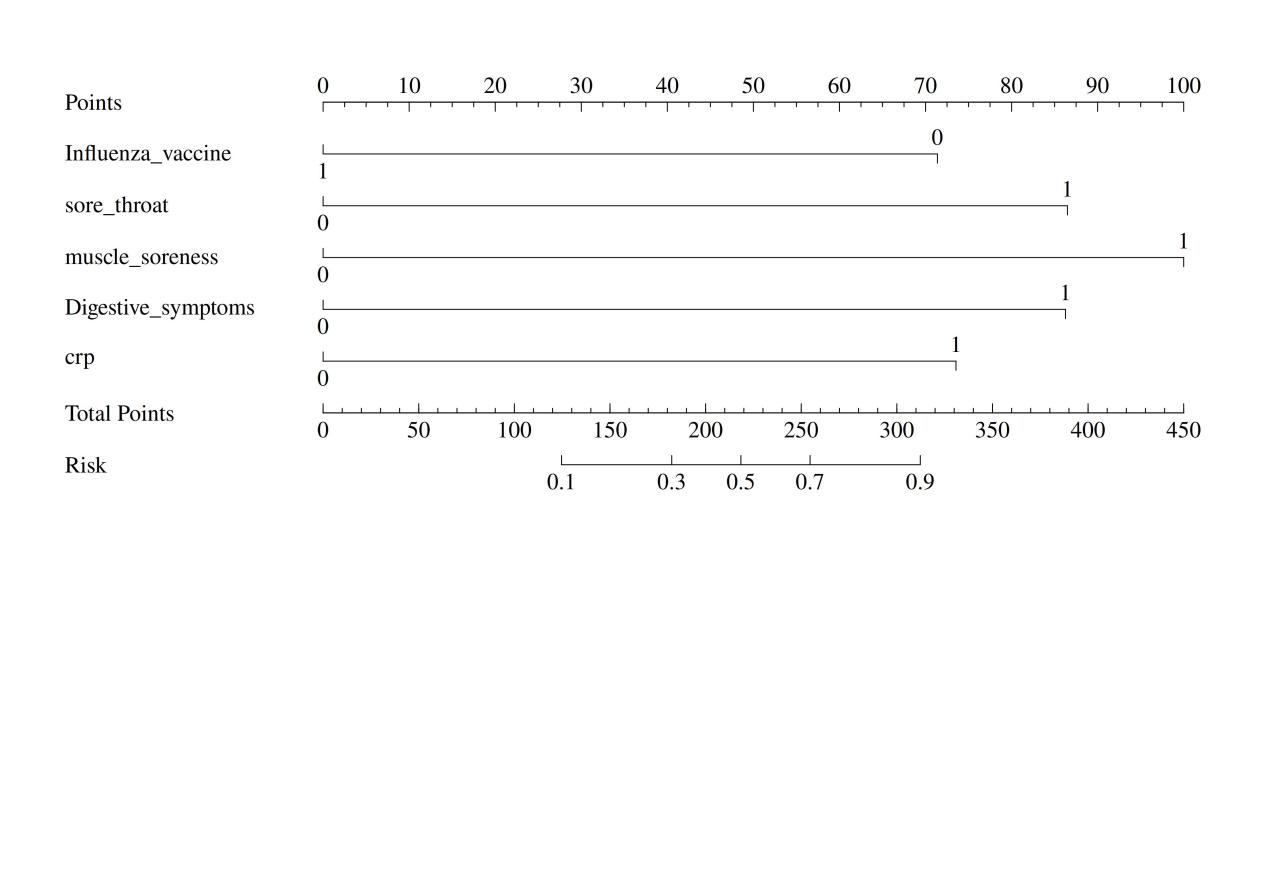
**

**Supplementary Material Figure 4** Receiver operating characteristic (ROC) curve of the baseline-only sensitivity analysis model, used to evaluate the discriminative ability of the model for predicting referral or hospitalization risk in children aged 6 months to 6 years with community-acquired influenza.

**
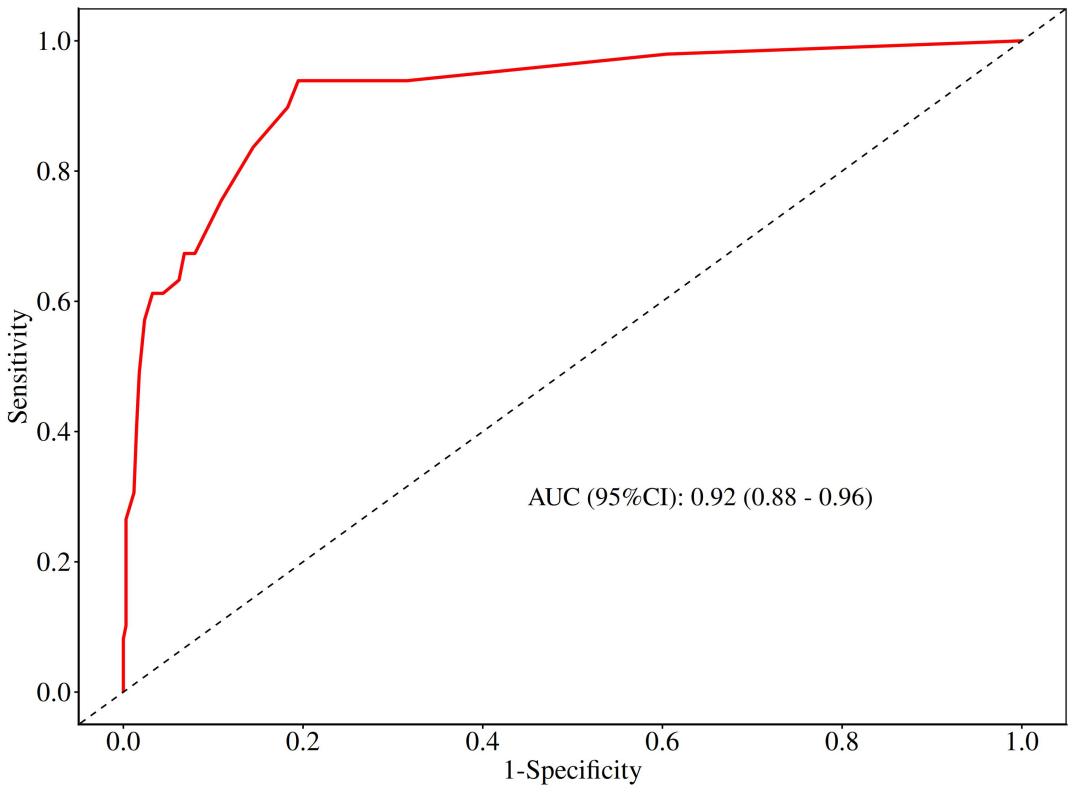
**

**Supplementary Material Figure 5** Calibration curve of the baseline-only sensitivity analysis model in the apparent dataset, used to assess the consistency between the model-predicted probability and the actual observed probability of referral or hospitalization in children aged 6 months to 6 years with community-acquired influenza.

**
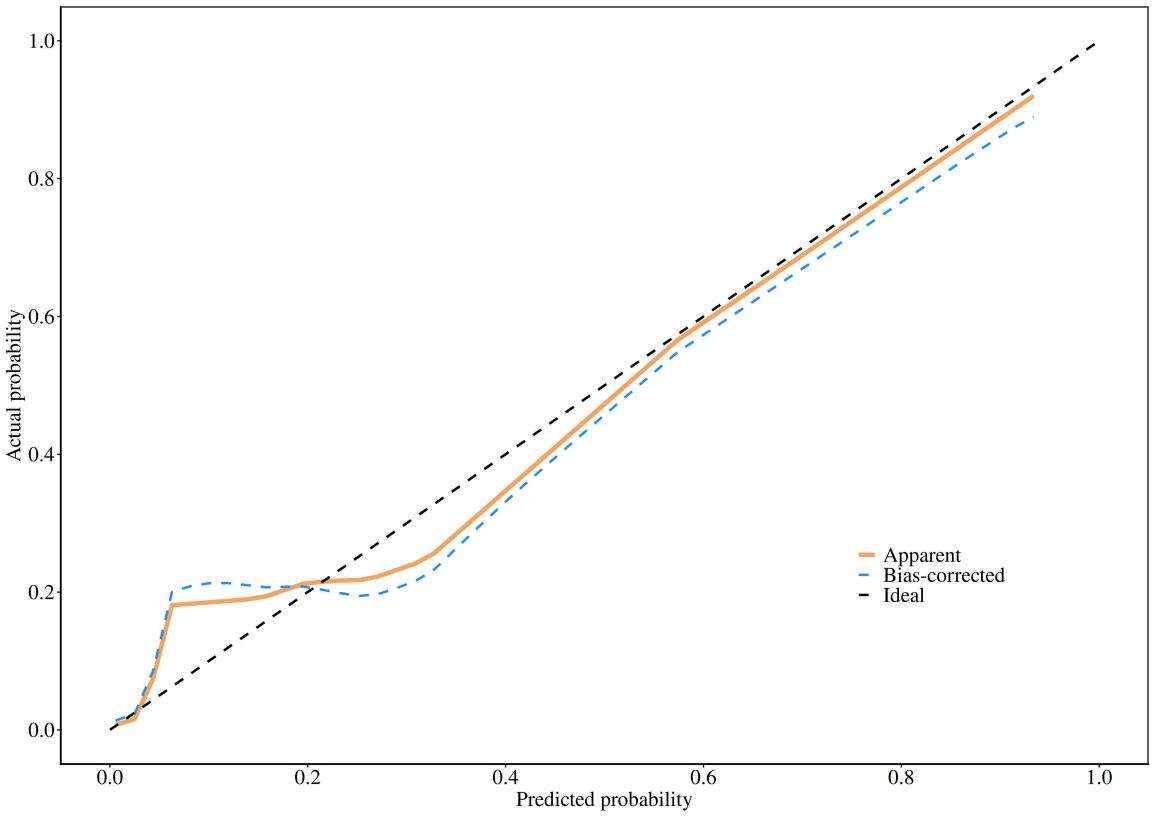
**

**Supplementary Material Figure 6** Decision curve analysis (DCA) curve of the baseline-only sensitivity analysis model, used to evaluate the clinical net benefit and clinical utility of the model for predicting referral or hospitalization risk in children aged 6 months to 6 years with community-acquired influenza across different threshold probabilities.

**
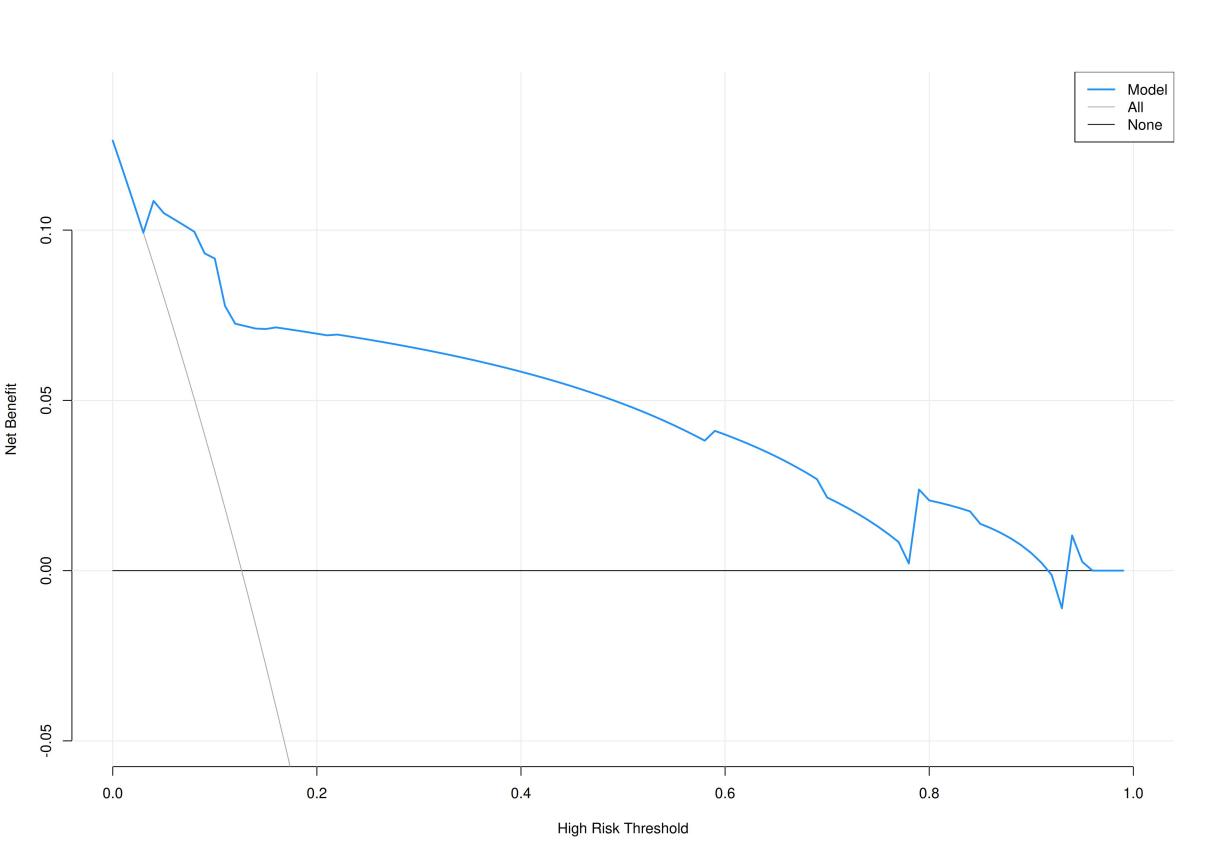
**

**Supplementary Material Figure 7** Variable importance ranking based on the SHapley Additive exPlanations (SHAP) method for the baseline-only sensitivity analysis model, which quantifies the relative contribution of each independent risk factor to the prediction of referral or hospitalization risk in children aged 6 months to 6 years with community-acquired influenza.

**
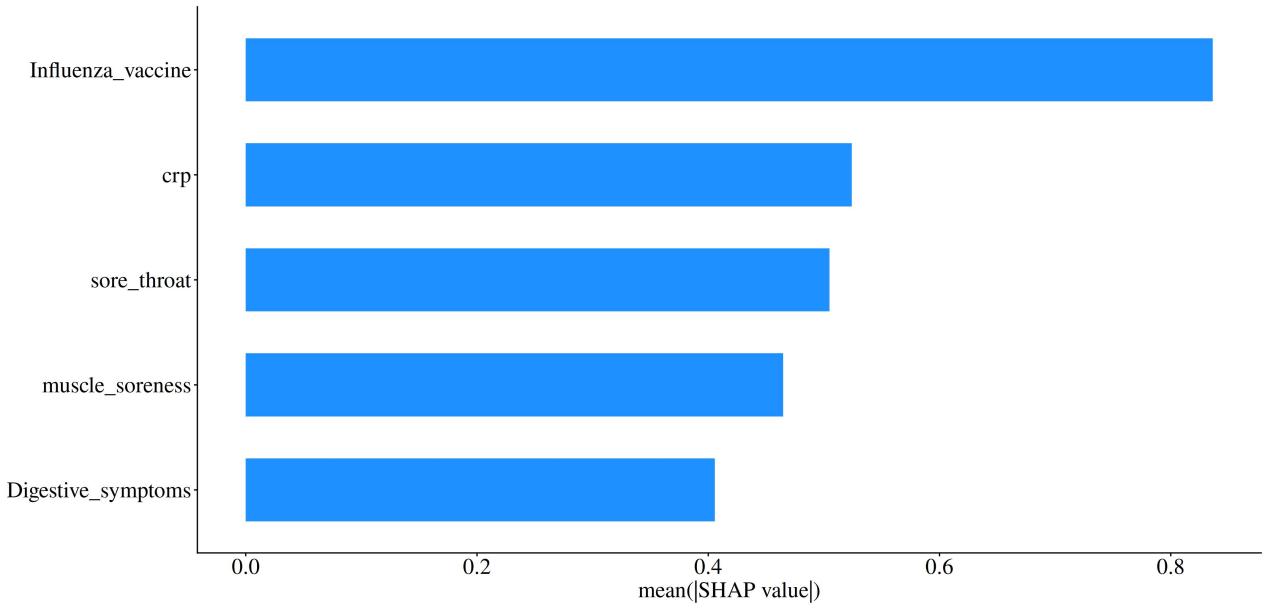
**

**Supplementary Material Figure 8** Calibration curve and calibration slope of the baseline-only sensitivity analysis model in the apparent dataset, used to further verify the goodness of fit of the model for predicting referral or hospitalization risk in children aged 6 months to 6 years with community-acquired influenza.

**
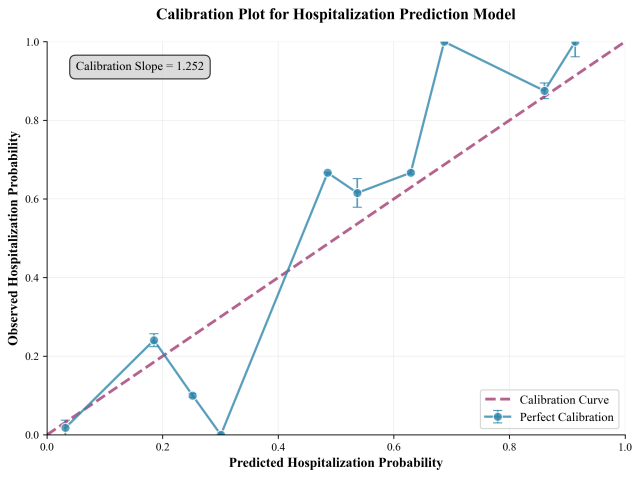
**

**Supplementary Material Figure 9** Receiver operating characteristic (ROC) curve of the baseline-only sensitivity analysis model after 1000 iterations of Bootstrap internal validation, used to verify the stability and discriminative ability of the model for predicting referral or hospitalization risk in children aged 6 months to 6 years with community-acquired influenza.


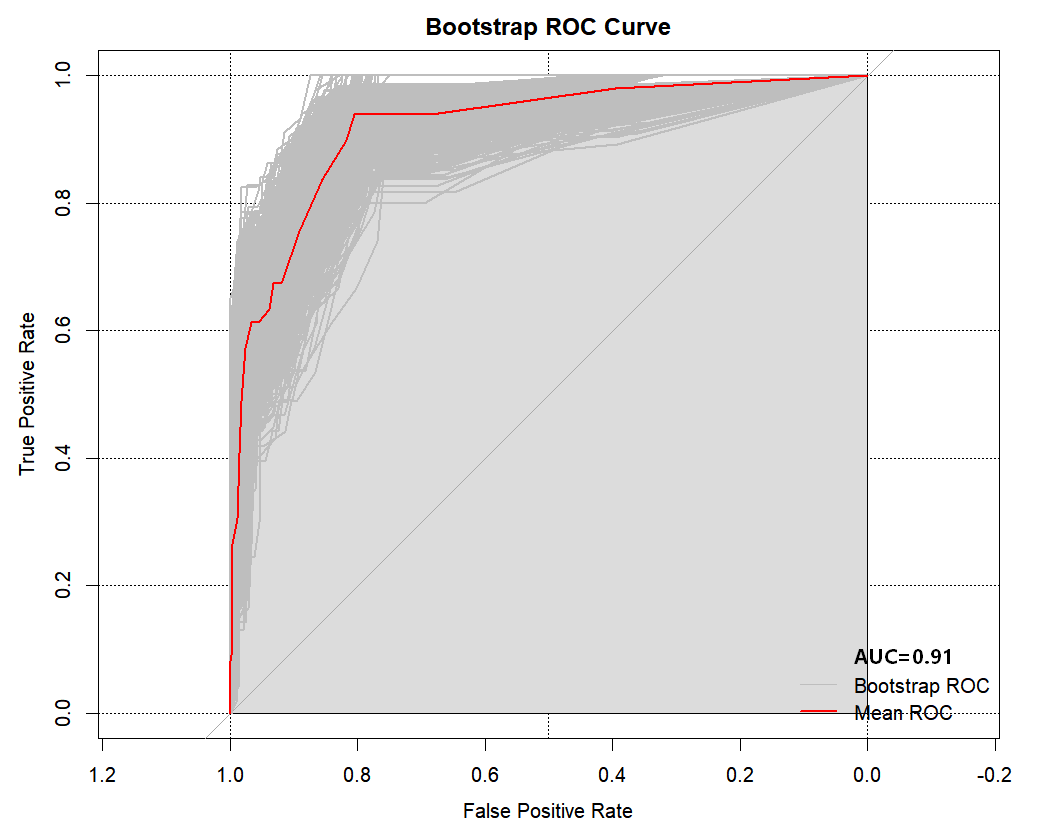


**Supplementary Material Figure 10** Calibration curves and calibration slopes of the baseline-only sensitivity analysis model after 1000 iterations of Bootstrap internal validation, used to verify the stability and goodness of fit of the model for predicting referral or hospitalization risk in children aged 6 months to 6 years with community-acquired influenza.

**
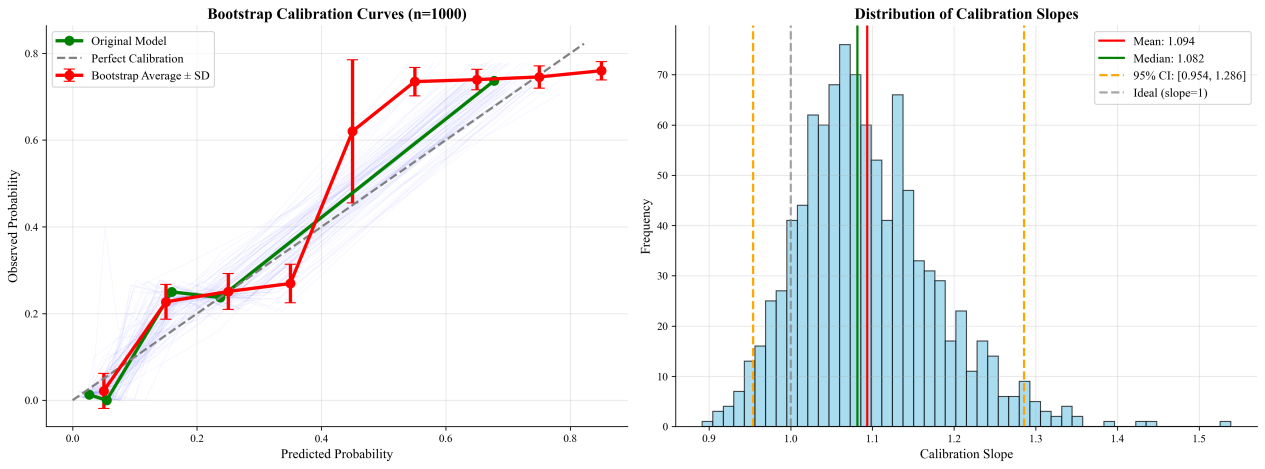
**
